# Toxic effect of organophosphate (chlorpyrifos) on hematological, biochemical, enzymatic and histological parameters in striped catfish (*Pangasianodon hypophthalmus*, Sauvage, 1878)

**DOI:** 10.1038/s41598-026-43759-3

**Published:** 2026-04-15

**Authors:** Bhagchand Chhaba, H. B. Dhamagaye, A. S. Pawase, E. Arun Goud, P. H. Sapkale, B. R. Chavan, S. J. Meshram, Priyanka Acharya, Soibam Ngasotter

**Affiliations:** 1 College of Fisheries, Dr. Balasaheb Sawant Konkan Krishi Vidyapeeth, Ratnagiri, 415629 Maharashtra India; 2 LSPN College of Fisheries, Dau Shri Vasudev Chandrakar Kamdhenu Vishwavidyalaya, Kawardha, 491995 Chhattisgarh India; 3 Regional Center, ICAR-Central Inland Fisheries Research Institute, Prayagraj, 211002 Uttar Pradesh India; 4 Fishery Survey of India, Mumbai, 400005 Maharashtra India

**Keywords:** Biochemical, Catfish, Chlorpyrifos, Hematology, Histology, toxicity, Biochemistry, Biomarkers, Environmental sciences, Physiology, Zoology

## Abstract

This study evaluated the impact of chlorpyrifos (CPF) on haematological parameters, enzymatic activity, biochemical indices, and tissue histology of striped catfish (*Pangasianodon hypophthalmus*). Fish were exposed to two sublethal concentrations of chlorpyrifos-20 EC (T1: 0.0106 mg L^−1^ and T2: 0.0212 mg L^−1^), along with a control group (0.0 mg L^−1^), for 45 days. The 96-h LC₅₀ value of chlorpyrifos-20 EC was determined as 0.106 mg L^−1^. Blood and tissue samples were collected on days 15, 30, and 45 for analysis. CPF exposure caused a significant decline in RBCs count, haemoglobin, haematocrit, total protein, albumin, and globulin, while WBCs count, mean corpuscular volume, mean corpuscular haemoglobin, serum glucose, and triglycerides were elevated. Serum enzymatic activities acid phosphatase (ACP), alkaline phosphatase (ALP) and aspartate aminotransferase (AST), alanine aminotransferase (ALT) and acetylcholinesterase (AChE) showed significant (*p* < 0.05) alterations, and antioxidant enzymes (SOD and catalase) were markedly reduced in treated groups. Histopathological alterations in the gill, liver, and kidney were more pronounced in T2 than in T1. Overall, CPF exposure induced severe haematological, biochemical, enzymatic, oxidative stress, and histological disturbances in striped catfish, indicating its potential risk to fish health and aquatic ecosystems.

## Introduction

In India, one method of managing agricultural pests is to employ pesticides such as insecticides, herbicides, and fungicides. But certain pests have developed resistance as a result of the extensive use of insecticides. Farmers frequently combine many different insecticides to manage the pest population to solve this issue. To combat pests that affect crops and homes, insecticides are commonly used in both urban and rural regions. In 2020, the global level of total pesticide utilization in agriculture was 2.7 million metric tons (MMT). Pesticides were applied to crops at a rate of 1.8 kg/ha globally. In 2020, the global trade in pesticides reached 7.2 MMT of formulated products, valued at USD 41.1 billion. With 3.7 MMT worth of pesticide exports in 2020, Asia led the world with a value of USD 16.1 billion^1^. Annual utilization of chemical and bio-pesticides in India was about 59,714 metric tons (MT) and Maharashtra tops in the utilization of pesticides with a value of 13,175 MT^2^. Organophosphorus (OP) pesticides are the most widely used synthetic chemicals for pest management that are harmful to aquatic creatures, particularly fish^3^.

Chlorpyrifos (O, O-diethyl O-3,5,6-trichloro-2-pyridyl phosphorothioate) is a broad-spectrum organophosphate pesticide that is widely used in agriculture all over the world to manage a wide range of insect pests. Due to its widespread application and enduring characteristics, chlorpyrifos often infiltrates aquatic habitats through agricultural runoff, spray drift, and drainage from treated fields, resulting in its ongoing presence in surface waters and sediments of freshwater ecosystems. This environmental prevalence raises ecological concerns due to the ongoing potential for exposure among non-target aquatic organisms, especially fish that reside in or migrate through contaminated waters^4^. In point of fact, chlorpyrifos residues have been documented in rivers and aquaculture systems, both of which are areas where agricultural activities are carried out in a significant manner, highlighting the need of comprehending the long-term ecological impact of this substance.

Chlorpyrifos works to eliminate pests by continuously inhibiting the function of acetylcholinesterase (AChE), which causes paralysis and eventually death^5^. Chlorpyrifos has the potential to block AChE at cholinergic synapses, which is the principal mechanism by which chlorpyrifos causes toxicity^6^. Concurrently, CPF inhibits the action of AChE by phosphorylating a serine residue at the active site of the enzyme^7^. This is a mechanism that has been demonstrated to be effective. Additionally, as stated, additional pathways that are known to be responsible for the effects of chlorpyrifos include increased lipid peroxidation and alterations in the redox status of cells^7^. The maximum quantity of chlorpyrifos recorded in rainwater collected from Hisar, Haryana, India, was up to 3 mg L^−1^^8^. With a half-life of 25.6 days (pH 7.0) in water^9^ and its harmful effects on both target and non-target aquatic organisms^10^, CPF is a strong candidate for toxicity studies. In toxicity studies, haematological indices are studied to evaluate the physiological status of fish. Variations in hematological parameters such as RBC, WBC and plasma and serum levels can result in histological abnormalities that may affect the liver, kidneys, gills, muscles, brain, and gut in many species of fish exposed to various pesticides^11^. Pathological or chemical stress can cause physiological disturbances, which can be forecast using the biochemical profile of blood^12^.

The elevated toxicity of CPF to fish and other aquatic species has made it a significant environmental contaminant. DNA damage and oxidative stress have been linked to this pesticide. Reactive oxygen species (ROS) are molecules that are highly reactive with one or more unpaired electrons that are generated by CPF metabolism. Histological studies offer insights into the site of action of immunotoxic chemicals, the extent of organ damage and the modes of action. The degree of tissue damage, injuries, and organ dysfunction indicates decreased development and fitness, survival, poor reproductive success, or enhanced vulnerability to pathogenic agents are all disclosed by histopathology.

As a result, the harmful effects of pollutants vary depending on the species, the environment, and the metabolites. Toxicological endpoints, such as physiological, behavioural, biochemical, immunological, or histological studies, can be used to identify these effects.

Fish are considered a cheap and rich source of protein with good nutritional value; however, due to their sensitivity to environmental changes, they are often used as bio-indicators for detecting the degree of pollution in aquatic environments^13^. The striped catfish (*Pangasianodon hypophthalmus*) is one of the most prevalent freshwater fish species. This fish was originally imported to India in 1997 from Bangladesh in the state of West Bengal^14^. Desired characteristics like faster growth, lower trophic level feeding habit and good feed conversion efficiency have laid a pathway for intensive culture of catfish in tanks, cages, ponds, etc., with better yield^15^. The present study attempted to examine the toxicological risk through assessments of haematological, biochemical, expression of enzyme activity and histopathological response of *P. hypophthalmus*. This evaluation was carried out in order to meet the crucial gap that has been identified. Our understanding of the consequences of pesticides at actual exposure concentrations and durations will be enhanced as a result of the findings from such thorough experiments, which will in turn inform both environmental protection regulations and aquaculture practices.

## Materials and methods

### Collection and maintenance of animals

The striped catfish fingerlings of uniform size (length 8 ± 1 cm, weight 4.8 ± 0.7 g) were acquired from a local supplier in Ratnagiri, Maharashtra, India and were acclimated in an FRP tank (500 L capacity) to experimental conditions for 15 days before starting the experiment. The experiment was conducted at the Wet Laboratory, College of Fisheries, Ratnagiri, Maharashtra, India. According to APHA^16^, the physicochemical properties of the tap water utilized in this investigation were temperature 27 ± 1.0 °C, pH 7.2 ± 0.14, dissolved oxygen 5.8 ± 0.25 mg L^−1^, and alkalinity 59.0 ± 2.34 mg L^−1^. Fish were fed with commercial feed (28% crude protein) (4% of b/w twice a day) throughout the experimental period. The fish were starved for one day before starting the experiment.

### Toxicant used

Locally purchased agrochemical, chlorpyrifos (O, O-diethyl-O-3,5,6-trichlor-2-pyridyl phosphorothioate) 20% EC (CAS No: 2921-88-2 or registration no. CIR-16,143/93-CHLORPYRIFOS (EC)-388) was used for the present study.

### Acute toxicity test

The acute toxicity test of chlorpyrifos (CPF) was carried out according to OECD^17^ in a static system by using a 50 L capacity glass aquarium. A total of five concentrations, viz. 0.09, 0.1, 0.11, 0.12 and 0.13 mg L^−1^ in triplicate, along with a control, were selected based on the range finding test (0.05, 0.1, 1.5, 2.0 and 2.5 mg L^−1^). In each tank, 10 fish having an average weight and length of 4.8 ± 0.7 g and 8 ± 1 cm, respectively, were stocked. The experimental duration was 96 h. The fish were starved during the experiment. Observations were taken at 6, 12, 24, 48 and 96 h respectively. In addition to the behavioral changes, the cumulative mortality percentage was recorded for 96 h. Dead fish were removed from the tank with the help of a scoop net. A probit analysis^18^ method was used to determine the LC_50_ value of CPF on *P. hypophthalmus*.

### Sublethal toxicity study

Sub-lethal studies were carried out to understand the hematological, biochemical, enzymatic activity and histological changes in the fish for 45 days with 15-day interval sampling. Sub-lethal toxicity test was performed according to a semi-static renewal system^17^. In all, two concentrations of CPF that are 1/10th (0.0106 mg L^−1^ – T1) and 1/5th (0.0212 mg L^−1^ – T2) of LC_50_ were taken for sublethal toxicity tests, including one control (0.0 mg L^−1^- C)^19^. A quantity of ten fish each from the acclimatized group was distributed randomly (CRD) to the experimental tanks in triplicate. Water was exchanged daily and a fresh chemical solution was added to it to keep the desired concentration levels. Round-the-clock aeration was provided to all the experimental units using an aerator. Feeding and other conditions were the same as acclimatization conditions.

### Collection of blood for the assay

For blood analysis, the test animal was randomly chosen from experimental tanks. Fishes were anesthetized using clove oil (Novelty Pharma Products, India, 50 µl L^−1^) and blood was drawn. Blood was collected from the posterior caudal vein using a 1.0 ml disposable insulin syringe and transferred immediately into EDTA-coated test tubes and later used for hematological examination. While the determination of biochemical parameters and enzyme activity, blood was transferred to a nonheparinized microcentrifuge tube. After centrifuging the serum at 3000 g for 15 min to separate them, they were stored at -20 °C until needed^20,21^.

### Hematological analysis

Total erythrocyte count (TEC/WBC) was performed using a Neubauer’s improved double hemocytometer with the help of RBC diluting fluid^22^. Erythrocyte (RBCs) was counted in the loaded haemocytometer chamber and the total number was recorded as ×10^6^ mm^−3^ Wintrobe mehod^23^. White blood cells (WBCs) or total leucocyte count (TLC) were counted using leucocyte dilution fluid (Qualigens) in a Neubauer’s counting chamber of a hemocytometer according to the Shaw method^24^. Blood haemoglobin content was estimated using the Cyanmethemoglobin method^25^ using Drabkin’s Fluid (Qualigens), while Snieszko’s micro-hematocrit method^26^ determines hematocrit (Hct). Standard formulas (Eqs. 1, 2) are used to estimate haematological indices of MCV and MCH^27^.

1 $${\mathrm{Mean}}\;{\mathrm{corpuscular}}\;{\mathrm{heamoglobin}}\;\left( {{\mathrm{MCV}}} \right)=\left( {{\mathrm{Hct}}/{\mathrm{RBC}}} \right) \times 10$$

2 $${\mathrm{Mean}}\;{\mathrm{concentration}}\;{\mathrm{heamoglobin}}\;\left( {{\mathrm{MCH}}} \right)=\left( {{\mathrm{Haemoglobin}}/{\mathrm{RBC}}} \right) \times 10$$

### Serum enzyme assay

Acid phosphatase (ACP), Alkaline phosphatase (ALP) and Aspartate aminotransferase (AST), Alanine aminotransferase (ALT) activities were estimated using the methods described by Reitman, S., and Frankel mehod^28^ and Walter, K., and Schutt^29^, respectively. The AChE activity assay was conducted using the Ellman et al.^30^ colorimetric method, using a cuvette with cholinergic iodide (0.015 M) and dithiobis nitrobenzoic acid (0.01 M) as substrates together with 0.1 M phosphate, pH 8.0. AChE activity was detected at 405 nm for 180 s.

### Assay of antioxidant enzymes

#### CAT activity

The CAT enzyme activity was estimated by measuring the breakdown of H_2_O_2_ using the method given by Luck^31^. This assay consists of 3 ml H_2_O_2_ phosphate buffer (consisting of phosphate buffer of 0.067 M and 2 mM H_2_O_2_) and tissue homogenate supernatant of 0.05 ml. Later change in absorbance was recorded at 240 nm using a UV–vis spectrophotometer for three minutes at 30 s intervals.

### SOD activity

SOD enzyme activity was determined using the method given by Marklund and Markalund^32^, which was based on the inhibition of pyrogallol auto-oxidation, and the value is represented as unit mg^−1^ protein. An ml of assay medium consists of 0.2 mM pyrogallol and 50 mM Tris–HCl buffer (pH 7.5). For 3 min, the pyrogallol autoxidation was monitored at 420 nm after adding various tissue homogenates from different treatments. A unit enzyme activity is described as the quantity of enzyme required to cause 50% inhibition of the autoxidation of pyrogallol under standard assay conditions.

### Serum biochemical assay

The level of glucose was estimated using an enzymatic colorimetric test by Mendel et al.^33^ Serum protein was estimated according to the Biuret method^34^. The methods of Doumas et al.^35^ and Bucolo and David^36^ were used to determine the albumin (Alb) and total triglyceride (TG) levels, respectively. Globulin content was calculated by subtracting albumin values from the total serum protein.

### Histological study

The kidney, gill and liver samples were collected from each concentration during sampling. After collecting, all the tissues were fixed in a 2 ml vial with 10% neutral buffer formalin (NBF) for 24–48 h. After fixation, the tissue samples were kept overnight in 70% ethyl alcohol.

Dehydration of the samples was done in graded alcohol, for processing. Sample blocks were prepared by embedding the tissues in paraffin and the blocks were then stored at 4 °C for 24 h. With the help of a microtome (Leica RM2255, USA), tissue Sect.  (5 μm) were prepared and then rehydrated in graded alcohol. The sections were subsequently stained using haematoxylin and eosin (H & E) and observed under a microscope (Carl-Zeiss-Promenade 1007745 Jena, Germany) using TUCSEN (Model-ISH1000) camera (TC capture software) attached to the microscope. Histopathological examinations were conducted as described by Bullock^37^.

### Statistical analysis

**Fig. 1:**
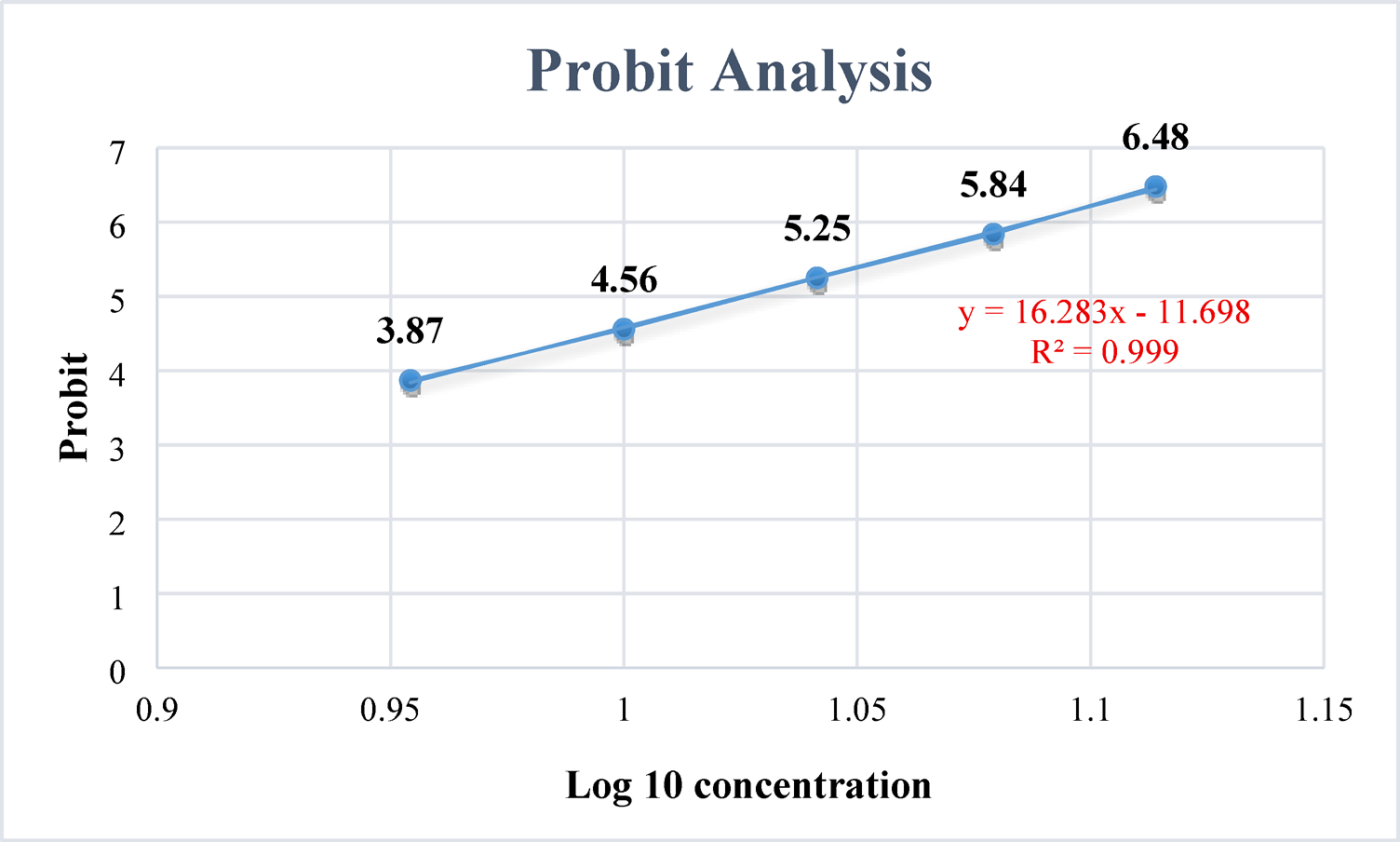
Linear relationship between mean probit mortality and log concentration of *P. hypophthalmus* fingerlings exposed to acute concentrations of chlorpyrifos for 96 h.

The LC_50_ value was estimated using probit analysis method^15^. To estimate regression parameters, the observed percentage kill was converted to probits. Figure 1 depicts typical relationships between log concentration and probit value. The regression equation is Y = a + bX + e, where a is the intercept, b is the slope of the line, and e is the error term. Sublethal testing, haematological, enzymatic activity, biochemical and physiological parameters of fingerlings of *P. hypophpthalmus* were analyzed statistically using One-way Analysis of Variance (ANOVA) and Tukey’s range test with the help of SPSS software (Version 16.0) to analyze significant differences and the homogeneity of variances among different experimental treatment groups.

## Results

### Median lethal concentration

The lethal concentration of chlorpyrifos for the striped catfish (*P. hypophthalmus)* was determined at chlorpyrifos levels ranging from 0.09 to 0.13 mg L^−1^. For 96 h, there was no mortality in the control (0 mg L^−1^). Probit analysis revealed that the median lethal concentration (Fig. 1), or the concentration required for 50% fish mortality, was 0.1060 mg L^−1^.

### Haematological parameters

Hematological studies revealed a significant decline in erythrocyte count (RBC), hemoglobin content (Hb), heamatocrit (Hct) in chlorpyrifos exposed groups at sublethal concentrations (T1–0.0106 mg L^−1^ and T2–0.0212 mg L^−1^) in a time-dependent manner compared to the control group (Table 1). By day 45, Haemoglobin (Hb) levels dropped by about 25% in the lower concentration and about 42% in the higher concentration compared to the control, suggesting increasing anaemia and a reduced ability to transfer oxygen. The T2 (0.0212 mg L^−1^) group RBC count dropped from 1.03 × 10⁶ mm⁻³ on day 15 to 0.39 × 10⁶ mm⁻³ on day 45, which is approximately 70–75% lower than that of the control group and suggests severe anaemia stress. In the T1 group (0.0106 mg L^−1^), the decline was less noticeable but still noteworthy. Packed cell volume (PCV/Hct) values significantly dropped in treated fish. By day 45, the highest CPF concentration caused a ~ 40% drop in PCV, which supported the reported drops in Hb and RBC counts and confirmed the development of hemodilution and anaemia.

**Table 1:** Hematological parameters of *P. hypophthalmus* exposed to different concentrations of chlorpyrifos for 45 days.

Parameters	15th Day			30th Day			45th Day		
	Control	T1	T2	Control	T1	T2	Control	T1	T2
Hb (g dL^−1^)	7.77±.09^a^	7.07±.09^b^	5.90±.15^c^	7.73±.02^a^	6.37±.08^b^	5.13±.12^c^	7.53±.08^a^	5.67±.12^b^	4.40±.11^c^
RBC (×10^6^/mm^3^)	1.42±.04^a^	1.13±.03^b^	1.03±.04^b^	1.4±.03^a^	0.87±.03^b^	0.54±.03^c^	1.4 ± 0.02^a^	0.61 ± 0.03^b^	0.39 ± 0.04^c^
WBC (×10^3^/mm^3^)	11.667 ± 0.088^c^	14.433 ± 0.067^b^	15.167 ± 0.088^a^	11.933 ± 0.088^c^	17.000 ± 0.057^b^	18.667 ± 0.088^a^	11.767 ± 0.145^c^	21.067 ± 0.176^b^	23.533 ± 0.033^a^
PCV (%)	17.95±.05^a^	15.17±.12^b^	13.33±.12^c^	17.67±.08^a^	14.67±.09^b^	11.57±.07^c^	17.87±.03^a^	13.20±.12^b^	10.67±.09^c^
MCV (fl.)	129.95 ± 4.62^a^	134.84 ± 4.47^a^	129.51 ± 5.79^a^	127.36 ± 1.99^a^	167.59 ± 4.77^c^	216.55 ± 10.51^b^	129.95 ± 1.89^a^	216.27 ± 11.83^b^	275.97 ± 24.64^b^
MCH (pg)	54.53 ± 1.62^b^	62.77±.88^a^	57.25 ± 2.28^ab^	55.12 ± 0.92^a^	72.71 ± 1.42^c^	95.89 ± 2.49^b^	53.69 ± 0.33^a^	92.75 ± 4.46^b^	113.36 ± 7.77^b^

Significant differences (*p* < 0.05) are indicated by different alphabetic superscripts.

In contrast, WBC, MCV, and MCH values increased as compared to the corresponding control groups. By day 45, WBC levels increased by approximately 79% in T1 and 100% in T2 relative to the control, suggesting that exposure to CPF triggered immunological and stress-related reactions. By day 45, the lower exposure group’s MCV had increased by about 67%, while the higher exposure group’s had increased by more than two times. In the same direction, MCH values increased significantly in each treatment group, reaching approximately 73% and 111% above control levels.

### Biochemical studies

Table 2 shows the biochemical profile of the fish upon exposure to various concentrations of CPF. Exposed groups had significantly lower levels of total serum protein, albumin, and globulin (*p* < 0.05), with the highest concentration resulting in reductions of approximately 66% (TP), 58% (ALB), and 78% (GLB) by day 45, indicating impaired protein metabolism and hepatic dysfunction. On the other hand, at the higher concentration by day 45, serum triglyceride and glucose levels increased significantly (*p* < 0.05), reaching approximately 55% and 62% above control values, respectively, indicating increased energy mobilisation and stress-induced metabolic disruption. No significant variations were observed in the control group throughout the experimental period.

**Table 2:** Biochemical parameters of *P. hypophthalmus* exposed to different concentrations of chlorpyrifos for 45 days.

Parameter	15th Day			30th Day			45th Day		
	Control	T1	T2	Control	T1	T2	Control	T1	T2
Total protein (g dL^−1^)	4.30 ± 0.06^a^	3.73 ± 0.08^b^	2.63 ± 0.09^c^	4.17 ± 0.19^a^	3.70 ± 0.25^b^	2.17 ± 0.09^b^	4.33 ± 0.08^a^	2.93 ± 0.08^b^	1.43 ± 0.08^c^
Albumin (g dL^−1^)	2.33 ± 0.12^a^	2.00 ± 0.06^a^	1.60 ± 0.06^b^	2.33 ± 0.09^a^	1.90 ± 0.15^a^	1.27 ± 0.09^b^	2.38 ± 0.02^a^	1.73 ± 0.09^b^	1.00 ± 0.10^c^
Globulin (g dL^−1^)	1.97 ± 0.17^a^	1.73 ± 0.08^a^	1.03 ± 0.03^b^	1.83 ± 0.18^a^	1.80 ± 0.35^a^	0.90 ± 0.12^a^	1.95 ± 0.1^a^	1.20 ± 0.06^b^	0.43 ± 0.03^c^
Triglyceride (mg dL^−1^)	59.87 ± 0.78^a^	69.53 ± 0.60^b^	80.17 ± 1.40^c^	60.67 ± 0.97^a^	75.83 ± 1.07^b^	88.40 ± 0.55^c^	61.87 ± 1.41^a^	83.00 ± 0.64^b^	95.90 ± 0.82^c^
Glucose (mg dL^−1^)	37.10 ± 0.92^a^	42.67 ± 0.87^b^	53.07 ± 0.65^c^	37.00 ± 0.82^a^	48.40 ± 0.65^b^	57.07 ± 0.68^c^	38.67±.99^a^	53.70 ± 1.19^b^	62.67 ± 1.14^c^

Significant differences (*p* < 0.05) are indicated by different alphabetic superscripts.

### Enzyme activity

**Fig. 2:**
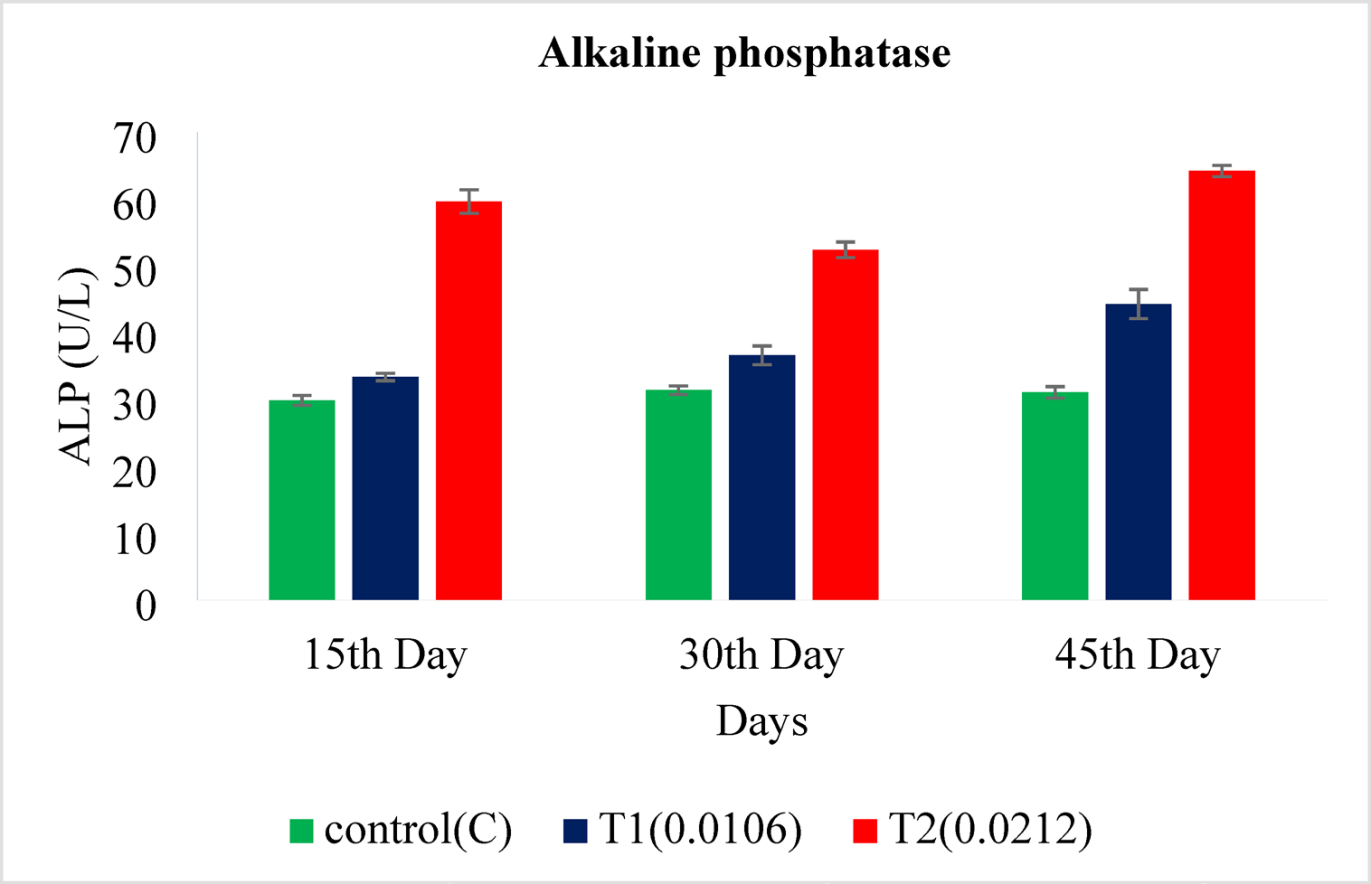
Alkaline Phosphatase (U/L) activity in the serum of the fish *P. hypophthalmus* at various periods of exposure to sublethal toxicity of chlorpyrifos. Data represents the mean ± SE.

**Fig. 3:**
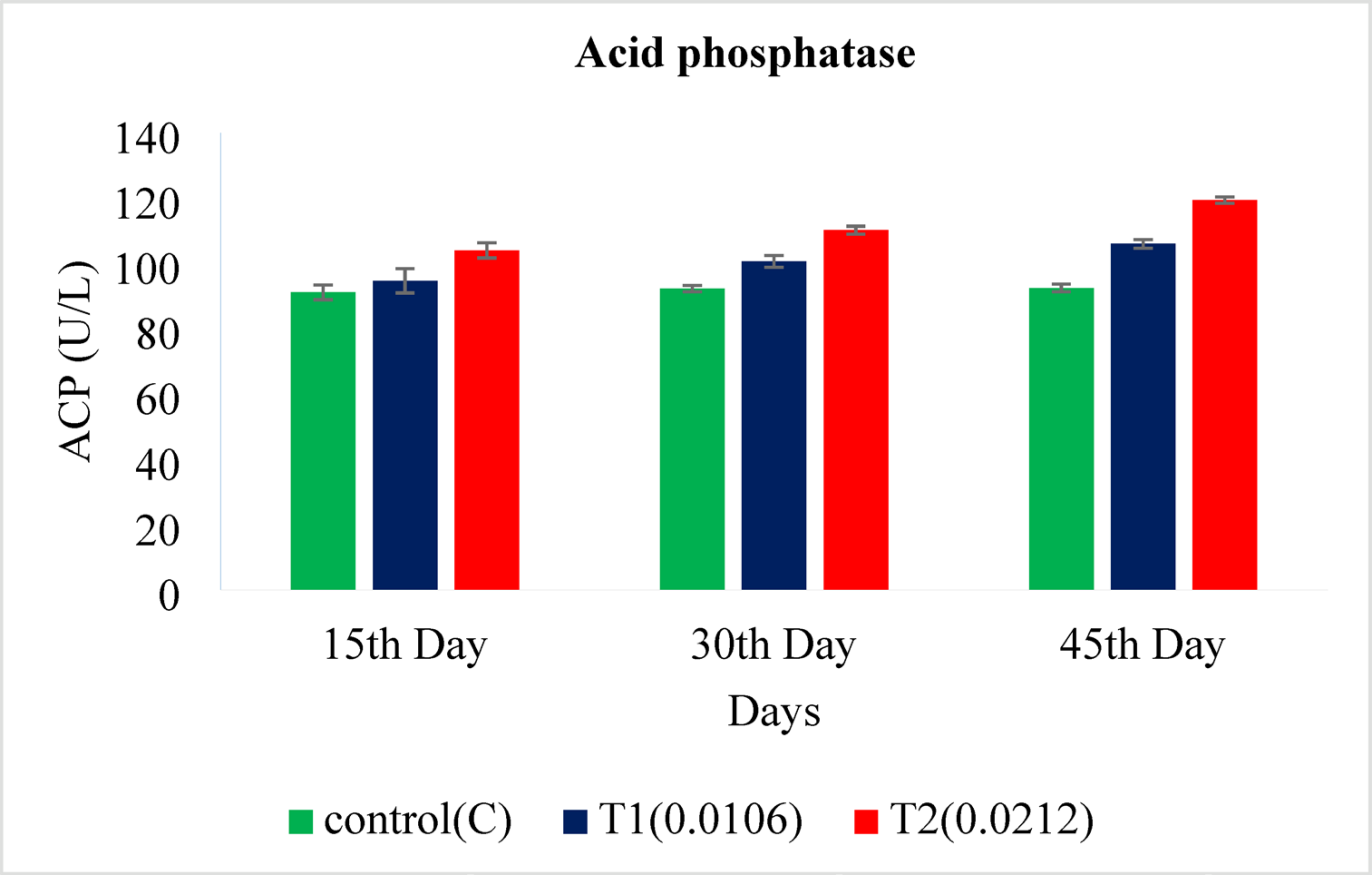
Acid phosphatase (U/L) level in the serum of the fish *P. hypophthalmus* at various periods of exposure to sublethal toxicity of chlorpyrifos. Data represents the mean ± SE.

**Fig. 4:**
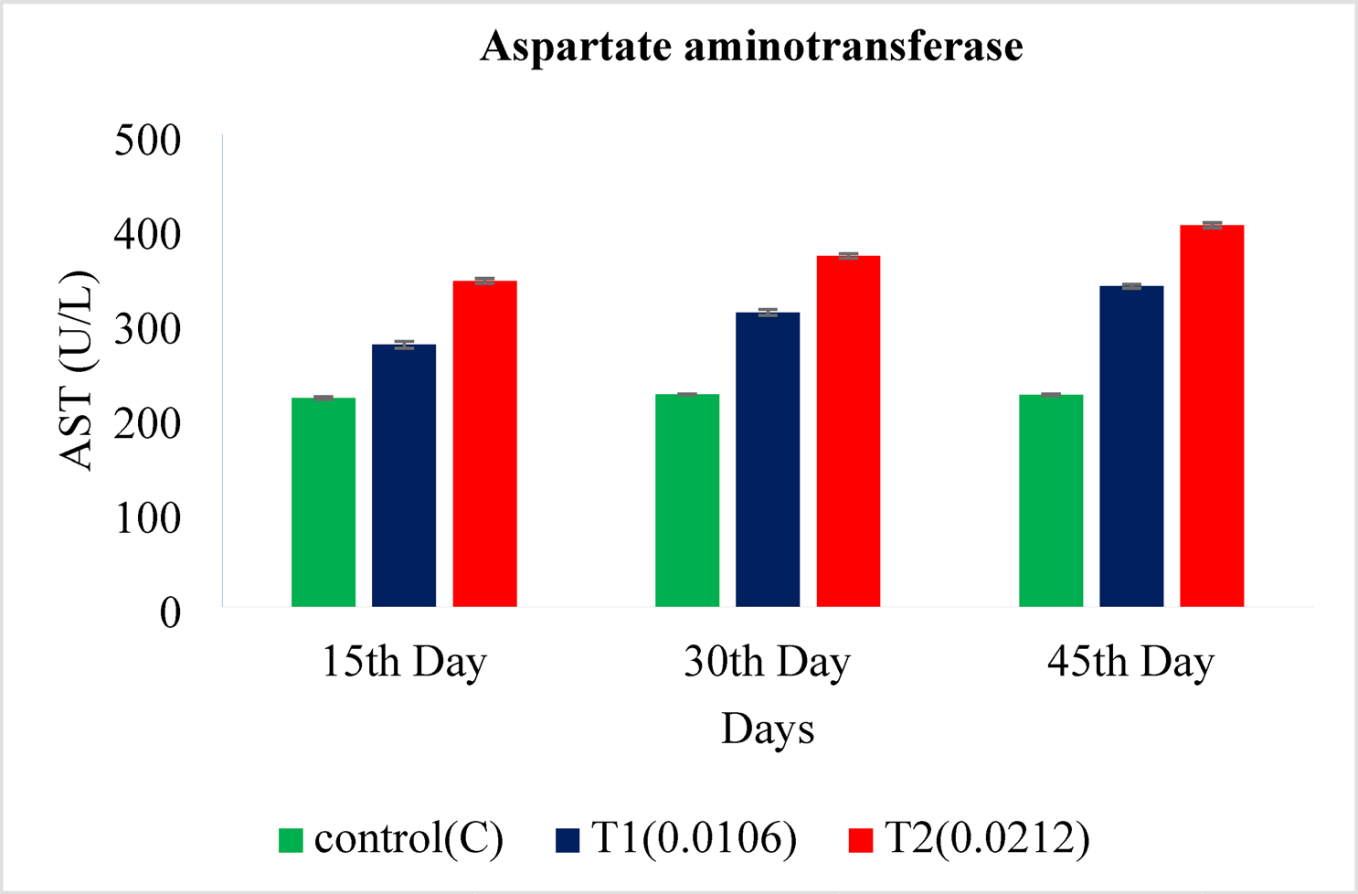
Aspartate aminotransferase (U/L) level in the serum of the fish *P. hypophthalmus* at various periods of exposure to sublethal toxicity of chlorpyrifos. Data represents the mean ± SE.

**Fig. 5:**
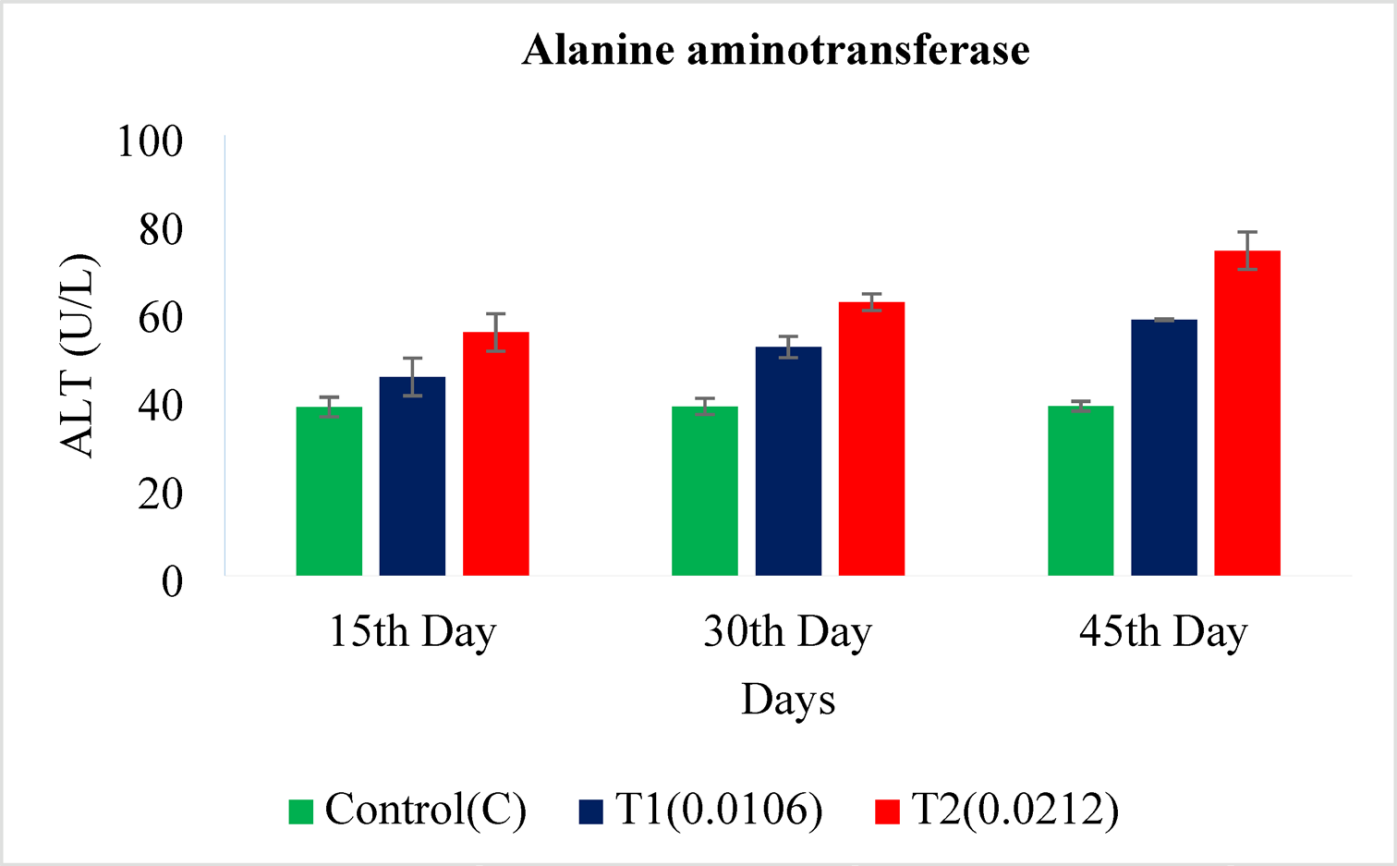
Alanine aminotransferase (U/L) level in the serum of the fish *P. hypophthalmus* at different periods of exposure to sublethal toxicity of chlorpyrifos. Data represents the mean ± SE.

**Fig. 6:**
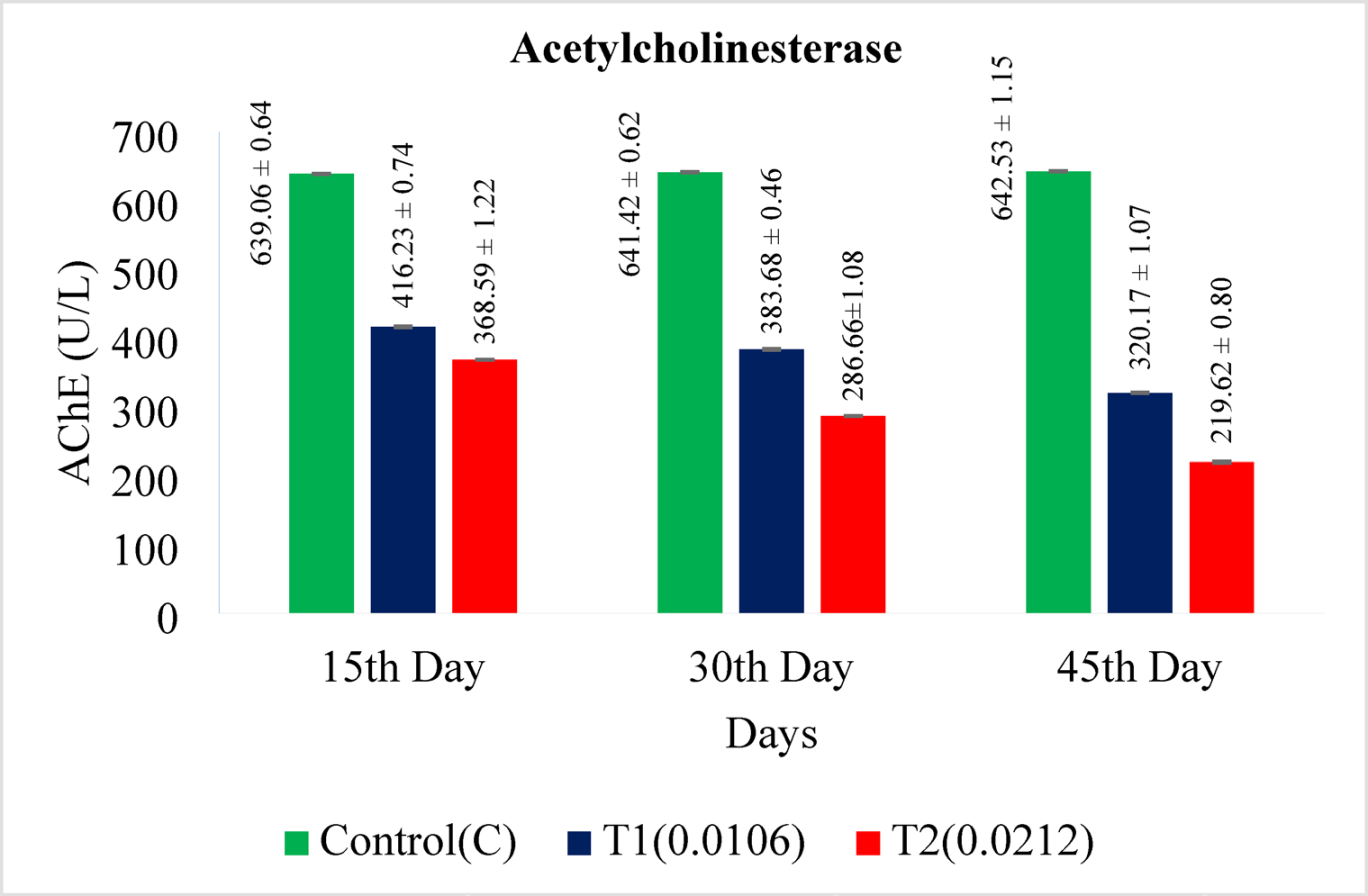
Acetylcholinesterase (U/L) level in the serum of the fish *P. hypophthalmus* at various periods of exposure to sublethal toxicity of chlorpyrifos. Data represents the mean ± SE.

The activity of serum enzymes, including ALP (Fig. 2), ACP (Fig. 3), AST (Fig. 4) and ALT (Fig. 5), were enhanced significantly (*p* < 0.05) in CPF treated fish (T1 and T2) at all experimental sampling days as compared to the control group. The highest elevations were observed at the higher concentration on day 45, suggesting increased lysosomal activity and membrane destabilisation. Aspartate aminotransferase (AST) and alanine aminotransferase (ALT) also demonstrated progressive and significant (*p* < 0.05) increases over the course of the exposure period. By day 45, the higher-dose group’s AST and ALT activities were roughly twice as high as those of the control group, indicating severe hepatocellular damage and enzyme leakage into the blood. AChE enzyme activity was measured in the serum of striped catfish after CPF exposure for 15, 30 and 45 days, as shown in Fig. 6. AChE activity significantly (*p* < 0.05) decreased with increasing doses of CPF and exposure time. The highest inhibition was observed on day 45 at the higher dose, confirming CPF-induced neurotoxicity.

### Antioxidant enzyme activities

The antioxidant enzymatic changes, namely SOD and CAT in gills, liver and kidney were shown in Tables 3 and 4, respectively. Upon exposure to CPF, CAT enzyme activity was inhibited and exhibited a significant decrease in all the organs. However, the results from the CPF exposed groups revealed a dose-dependent reduction in SOD enzyme activity in the examined organs. In general, significant changes in antioxidant enzymatic activity, namely CAT and SOD in gills, liver and kidney were observed in CPF exposed groups in comparison to the control.

**Table 3:** Variation in activity of SOD (unit mg^−1^ protein) in gill, liver and kidney of *P. hypophthalmus* on exposure to chlorpyrifos for 45 days.

Organ/ Tissue	15th Day			30th Day			45th Day		
	Control	T1	T2	Control	T1	T2	Control	T1	T2
Gill	45.236 ± 0.613^a^	41.773 ± 0.125^c^	38.658 ± 0.374^b^	45.574 ± 0.476^a^	39.966 ± 0.074^c^	36.516 ± 0.167^b^	45.189 ± 0.421^a^	39.171 ± 0.059^c^	33.207 ± 0.086^b^
Liver	36.86 ± 0.006^a^	34.287 ± 1.037^b^	30.097 ± 0.093^b^	36.202 ± 0.219^a^	31.615 ± 0.105^c^	29.225 ± 0.294^b^	36.764 ± 0.001^a^	29.857 ± 0.107^c^	27.437 ± 0.186^b^
Kidney	40.911 ± 0.111^a^	39.736 ± 0.127^c^	39.109 ± 0.072^b^	41.675 ± 0.068^a^	39.382 ± 0.074^c^	36.689 ± 0.499^b^	41.223 ± 0.054^a^	38.013 ± 0.585^c^	34.176 ± 0.632^b^

Significant differences (*p* < 0.05) are indicated by different alphabetic superscripts.

**Table 4:** Variation in activity of Catalase (unit mg^−1^ protein) in gill, liver and kidney of *P. hypophthalmus* on exposure to chlorpyrifos for 45 days.

Organ/ Tissue	15th Day			30th Day			45th Day		
	Control	T1	T2	Control	T1	T2	Control	T1	T2
Gill	6.05 ± 0.419^a^	4.04 ± 0.269^b^	3.93 ± 0.612^b^	5.98 ± 0.889^a^	3.27 ± 0.058^b^	2.75 ± 0.162^b^	6.02 ± 0.412^a^	2.89 ± 0.035^c^	1.41 ± 0.095^b^
Liver	11.52 ± 0.795^a^	8.91 ± 0.581^b^	8.17 ± 0.185^b^	11.18 ± 1.21^a^	8.38 ± 0.271^ab^	6.58 ± 0.206^b^	11.55 ± 0.874^a^	7.39 ± 0.867^b^	5.31 ± 0.296^b^
Kidney	13.21 ± 0.142^a^	8.57 ± 0.596^c^	8.17 ± 0.797^b^	13.83 ± 0.26^a^	7.13 ± 0.136^c^	5.83 ± 0.358^b^	13.68 ± 0.874^a^	6.74 ± 0.434^c^	4.22 ± 0.186^b^

Significant differences (*p* < 0.05) are indicated by different alphabetic superscripts.

### Histological studies

#### Histological alterations of gill tissue

**Fig. 7:**
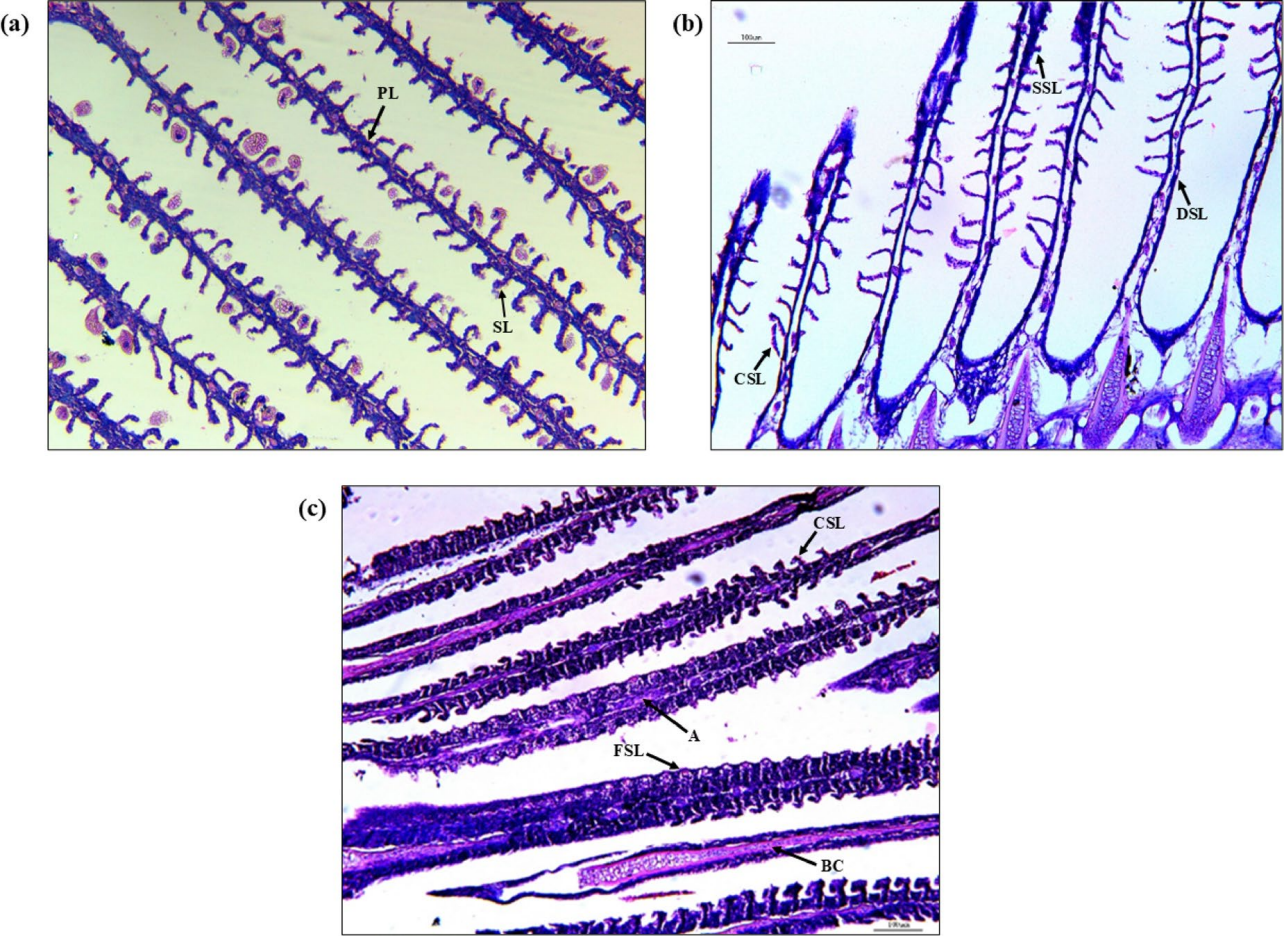
(**a**–**c**) On 45th day sub-lethal effect of chlorpyrifos exposure on the histological alteration in the gill tissue of stripped catfish (*P. hypophthalmus*). (**a**) Control 0.0 mg L^−1^ showing normal primary lamellae (PL), secondary lamellae (SL) and epithelium cell (Ep). (**b**) 0.0106 mg L^−1^ CPF exposed group showing shorting of secondary lamellae (SSL), curling of secondary lamellae (CSL) and degeneration of secondary lamellae (DSL). (**c**) 0.0212 mg L^−1^ CPF exposed group exhibiting collapsed secondary lamellae (CS), blood congestion (BC), massive fusion of SL (FSL) and aneurysms (A). (10× magnification and bars = 100 μm). (H&E stain and transverse section of gill tissue).

In the gills, no significant histological changes were recorded in the control groups (Fig. 7a). Different levels of histological alterations in gill tissues were observed 45 days after sublethal CPF exposure. In the T1 (0.0106 mg L^−1^) group, less significant changes were documented, which included shortening of secondary lamellae, curling of secondary lamellae (CSL) and degeneration of secondary lamellae (Fig. 7b). At a CPF exposure concentration of 0.0212 mg L^−1^ (T2), significant changes were observed, including collapsed secondary lamellae (CS), blood congestion (BC), massive fusion of SL (FSL) and aneurysms (A) (Fig. 7c).

#### Histological alterations of liver tissue

**Fig. 8:**
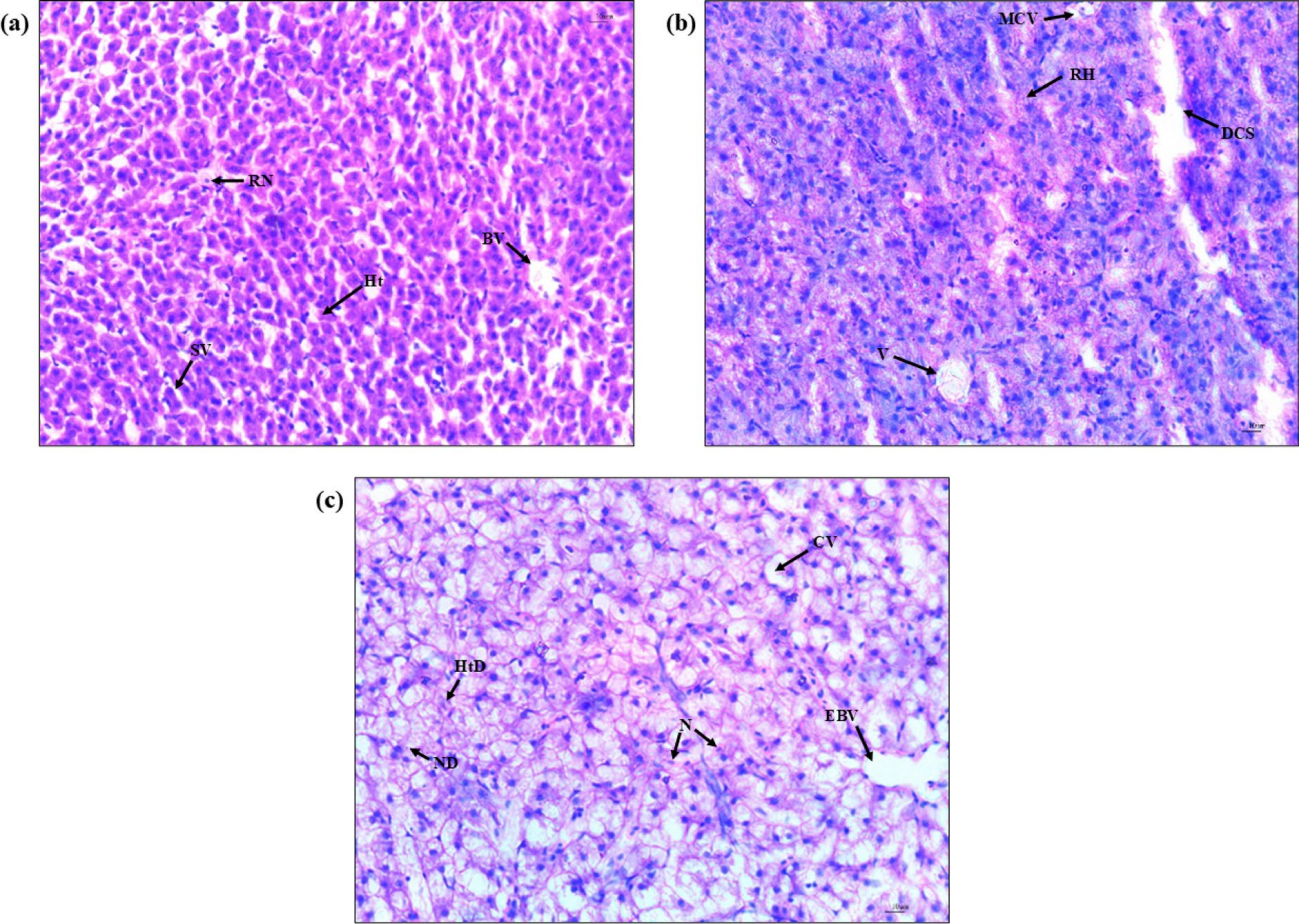
(**a**–**c**) On 45th day sub lethal effect of chlorpyrifos (CPF) exposure on the histological alteration in the liver tissue of stripped catfish (*P. hypophthalmus*). (**a**) Control (0.0 mg L^−1^) demonstrating normal architecture of hepatic cells (Ht), sinusoids vessels (SV), blood vessels (BV) and rounded nucleus (RN). (**b**) In 0.0106 mg L^−1^ CPF exposed group showing enlarged nuclei (EN), mild cytoplasmic vacuolation (MCV), dilation congestions in sinusoids (DCS), vacuolation (V) and ruptured hepatocytes (RH). (**c**) In 0.0212 mg L^−1^ CPF exposed group showing enlargement of blood vein (EBV), degeneration of hepatocytes cells (HtD), cytoplasmic vacuolation (CV), nuclear degeneration (ND) and necrosis (N). (40× magnification and bars = 10 μm). (H&E stain and transverse section of liver tissue).

No histological alterations were noted in the liver tissue of fish from the control group (Fig. 8a). The morphological alterations in liver structure manifest gradually with increasing concentrations. However, significant histological changes were observed in the liver tissue of fish in T2, including tissue enlargement, blood vein (EBV), degeneration of hepatocytes cells (HtD), cytoplasmic vacuolation (CV), degeneration of the nucleus (ND) and necrosis (N) (Fig. 8c). Mild cytoplasmic vacuolation (MCV), Dilation congestion in sinusoids (DCS), vacuolation (V) and ruptured hepatocytes (RH) (Fig. 8b) were observed in the T1 treatment group.

#### Histological alterations of kidney tissue

**Fig. 9:**
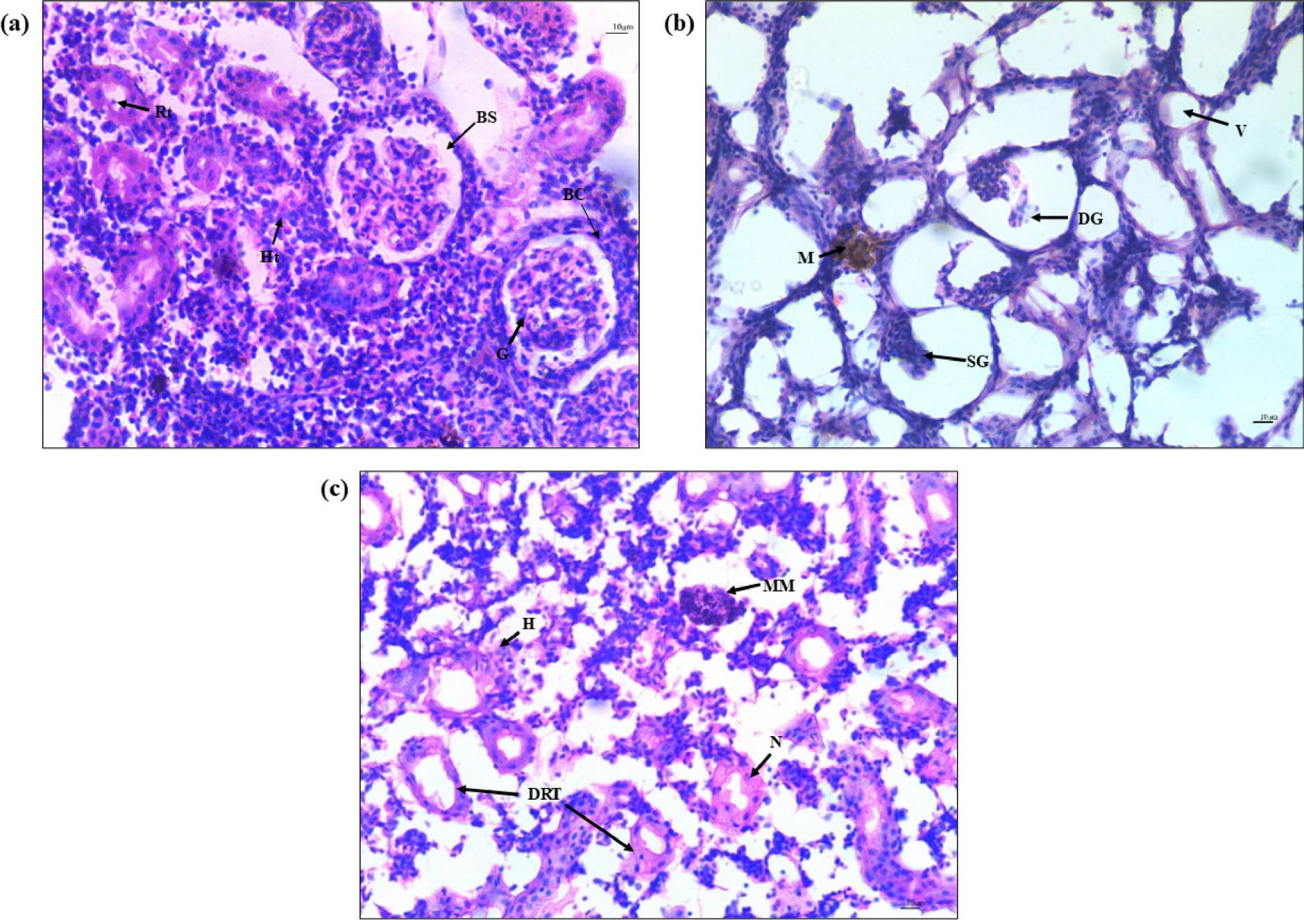
(**a**–**c**) On 45th day sub lethal effect of chlorpyrifos exposure on the histological alteration in the kidney of stripped catfish (*P. hypophthalmus*). (**a**) Control (0.0 mg L^−1^) showing normal architecture of renal tubules (RT), Bowman’s capsule (BC), glomerulus (G), hematopoietic tissue (Ht) and Bowman’s space (BS). (**b**) 0.0106 mg L^−1^ CPF exposed group exhibiting glomerulus shrinkage (SG), degeneration glomerulus (DG), mild vacuolation (V) and malanomacrophage (M). (**c**) 0.0212 mg L^−1^ CPF exposed group showing degeneration renal tubule (DRT), massive malanomacrophage (MM), tubular necrosis (N) and hyperplasia (H). (40× magnification and bars = 10 μm). (H&E stain and transverse section of kidney tissue).

The structure of the kidney in control fish, including Bowman’s capsules, renal tubules, glomerulus, hepatic tissue and Bowman’s space, showed no abnormalities (Fig. 9a). Fish treated in T1 (0.0106 mg/L) CPF exposed group showed glomerulus shrinkage (SG), degeneration glomerulus (DG), mild vaculation (V) and malanomacrophage (M) (Fig. 9b). In the T2 group, degeneration of renal tubule, massive malanomacrophage (MM), tubular necrosis and hyperplasia were noted (Fig. 9c). The findings emphasize the dose-dependent effect of CPF exposure on the renal histology of *P. hypophthalmus*.

## Discussion

The results of this study demonstrated that chlorpyrifos (CPF) is highly harmful to fish, with a 96-hour LC_50_ value of 0.106 mg L^−1^ for *P. hypophthalmus* fingerlings. A comprehensive review of toxicity data reveals varying 96-hour LC_50_ values in different fish species, underscoring potential species-specific responses to pesticides. Notably, previous studies report LC_50_ values of 0.165 mg L^−1^ for *Clarias batrachus*^38^, 46.8 µg L^−1^ for *Oreochromis niloticus*^39^, and 0.44 mg L^−1^ for *Cirrhinus mrigala*^40^. The various species described above may be more or less hazardous to certain pesticides depending on how they are absorbed, accumulated, biotransformed, and excreted. Acute toxicity is affected by test procedures, type of toxicant and species, which may explain the variations observed in different studies^41^. These variations underscore the complex interplay of biological and environmental factors influencing pesticide toxicity^42^.

It is critical to recognize the multidimensional character of pesticide toxicity, as aquatic creatures’ responses can be impacted by a variety of internal and extrinsic variables. This understanding enhances the contextual interpretation of LC_50_ values and reinforces the importance of considering the specificities of each species in ecological risk assessments related to pesticide exposure.

A study of haematological characteristics provides a better understanding of routine metabolic levels and toxicological impacts^43^. It’s observed that the RBC count, Hct and Hb levels declined drastically in the CPF treated fish. The results obtained are in parallel with the conclusions of^44–46^. Reductions in RBC count, Hct, and Hb levels could indicate anemia, which may be caused by inhibition of erythropoiesis, haemosynthesis, or osmoregulatory dysfunction, or by enhanced erythrocyte destruction in the hematopoietic organ^47^. The decrease in RBC number in chlorpyrifos-treated fish indicated that chlorpyrifos led to poor osmoregulation and gill injury. The decrease in haemoglobin content under sublethal treatment might have resulted from hemoglobinization or the shrinking of RBC caused by the toxic impact of chlorpyrifos on erythropoietic tissue. This haematological failure is mechanistically supported by the observed gill histopathology, including epithelial lifting and lamellar fusion, which reduces respiratory surface area and impairs gas exchange and osmoregulation. Chronic hypoxic stress resulting from gill dysfunction likely accelerates erythrocyte turnover and inhibits the transport of oxygen. Liver histology revealed hepatocellular degeneration and sinusoidal disruption, indicating impaired hepatic support for erythropoiesis and exacerbating the reported anaemic condition^48,49^.

In the present study, leukocyte counts increased significantly (*p* < 0.05) with increasing chlorpyrifos concentrations. The increase is due to the hypersensitivity of leukocytes to pesticide. The higher production of these leukocytes may be an immunological response of the fish to chlorpyrifos. These results are in agreement with various studies that show an increase in leucocytes in different fish species like *Labeo rohita*^45^, *O. mykis*^46^ and *Cyprinus carpio*^50^ exposed to chlorpyrifos. The increase in leukocytes in this study seems to indicate leukocyte hypersensitivity to the pesticide, and these haematological changes might have resulted from the fish’s immunological response to produce more leucocytes induced by chlorpyrifos.

The current study also noted alterations in blood cell indices, including MCH and MCV. This might be a result of the fact that they have a high sensitivity and can alter fish homeostasis irreversibly. Changes in these indices are directly correlated with changes in packed cell volume, haemoglobin concentration, and RBC count. Similar findings were reported in *Pseudetroplus maculatus*^51^ and *Oreochromis mossambicus*^52^ when exposed to chlorpyrifos and fenvalerate, respectively.

In conclusion, the haematological changes noted in this study give important insights into chlorpyrifos’ harmful effects on fish, altering both erythrocyte and leukocyte parameters. The results are consistent with earlier studies, reinforcing the sensitivity of these hematological indices as indicators of stress and potential toxicity in aquatic organisms.

The physiological disturbances that might occur in organisms because of pathological or chemical stress can be forecasted with the help of the blood’s biochemical profile. A hyperglycaemic condition was observed in fish exposed to chlorpyrifos compared to the control group, as indicated by elevated blood serum glucose levels. These elevations in glucose levels observed in chlorpyrifos treated groups may be attributed to processes such as glycogenolysis and gluconeogenesis. Similar results were observed in response to chlorpyrifos treatment in the blood serum of *Salmo trutta caspius*^21^ and *Clarias batrachus*^53^. In the CPF treated groups, serum protein, albumin and globulin levels were drastically reduced in comparison to the control group, demonstrating that CPF exhibits stress effects. Fish exposed to chlorpyrifos may have decreased levels of albumin and globulin because of the decrease in serum protein levels. The stress-mediated mobilisation of these substances to meet an increased demand for energy by fish to contend with the adverse conditions exposed to the toxicant may also be responsible for the significantly (*p* < 0.05) decreased serum protein, albumin and globulin levels in treated fish exposed to chlorpyrifos. Decreased protein levels may be seen in starvation and malabsorption and malnutrition^40^. Similar findings were observed by different authors^21,44,53^. Exposure to chlorpyrifos led to a significant increase in the triglyceride levels in the serum of fish. The elevation in serum triglycerides can be attributed to inhibition of the lipase enzyme or membrane damage by oxidative stress^38^. Similar findings were also reported in magur by Narra et al.^38^ and in *H. fossilis* by Prakash^54^, following exposure to chlorpyrifos. We also conclude that serum triglyceride levels have increased and this may be due to the hepatocellular tissues being damaged caused by CPF exposure.

The reprogramming of metabolic processes that is driven by stress is reflected in the rise of serum glucose and triglycerides. The mobilisation of carbohydrates and lipids is likely an adaptive response that occurs when a toxicant is exposed for an extended period of time^55^. This reaction is done in order to satisfy the higher energy demands involved with detoxification, antioxidant defence, and tissue repair^49^. This metabolic shift implies that energy resources are being diverted away from normal physiological maintenance, which is consistent with the cumulative nature of the toxic response that was observed over the course of the 45-day exposure.

Finally, based on consistent results across many fish species, the thorough analysis of the blood biochemical profile reveals a clear understanding of the stress-inducing effects of chlorpyrifos on the test fish. These results contribute to our understanding of the diverse impacts of chlorpyrifos on metabolic pathways and underscore the importance of considering these effects in the broader context of environmental stress in aquatic ecosystems.

ALT and AST are considered significant stress indicators and are frequently used in diagnosing fish diseases and identifying tissue damage caused by environmental contamination^56^. Hepatocellular damage or liver necrosis caused by exposure to chlorpyrifos is indicated by the increase in the activity of both enzymes. The changed enzyme activity may also point to cardiac damage, brain damage, or muscle dystrophy as a result of cellular deterioration following exposure to chlorpyrifos^52^. Similar increases in AST and ALT were observed previously chlorpyrifos treatment in the plasma of *Cyprinus carpio*^57,58^, and serum of Caspian trout^21^. ALP and ACP are important liver enzymes associated with detoxification, phosphate hydrolysis, membrane transport, and are also good biomarkers of stress in biological systems^57^. The current investigation found that exposure to sublethal concentrations of chlorpyrifos increased alkaline phosphatase (ALP) activity and acid phosphatase (ACP) activity in *P. hypophthalmus*. The same effects were documented in *Caspian trout*^21^, *Gambusia affinis*^59^ and *Oreochromis niloticus*^60^ for ALP and ACP, respectively on expose to chlorpyrifos. Alkaline phosphatase (ALP) plays an important role in transporting phosphorylated intermediates across cell membranes and in carbohydrate metabolism^61^. Similarly, hepatocellular damage may be the reason for increased acid phosphatase activity observed in fish exposed to chlorpyrifos^59^. Toxic agents can induce biochemical changes in the liver, leading to abnormalities in serum levels of ALT, AST, and ALP. These changes in serum levels can serve as indicators of toxic exposure. Similarly, exposure to sublethal concentrations of CPF leads to various severe morphological degenerations in the kidney, including tubular lumen narrowing, Bowman’s capsule contraction and renal epithelial cell necrosis. Thus, the enhancement in the activities of aminotransferase enzymes in the serum of *P. hypophthalmus* could be attributed to the secretion of enzymes from the liver cytosol into the bloodstream as a result of liver damage caused by chlorpyrifos intoxication.

The cholinesterase enzyme is necessary for the normal functioning of the nervous system and inhibiting this enzyme is the most important mechanism of organophosphate compounds. Fish exposed to chlorpyrifos showed decreased AChE activity in the blood, indicating the adverse effects of chlorpyrifos on serum AChE activity. Similar findings of decreased plasma/serum AChE activity have been documented in various fish species, such as *Rutilus caspicus*^62^, *M. nipponense*^63^, and *C. carpio*^58^, when exposed to various organophosphorus pesticides. Continuous stimulation of electrical signals and subsequent impairment in nervous system function have resulted from the accumulation of free acetylcholine at the nerve endings, which is attributed to the reduction of AChE.

In one word, the research emphasizes the extensive effects of chlorpyrifos on biochemical indicators, including enzymes linked to nervous system activity and liver function in *P. hypophthalmus.*

Oxidative stress is induced by organophosphate compounds, which alter the antioxidant status in organisms. According to the current study, the SOD and CAT antioxidant enzyme activity decreased in the gill, liver and kidney tissues. Therefore, it’s worthwhile to evaluate the oxidative stress and regulation of antioxidant enzymes in the gills, liver, and kidney resulting from exposure to chlorpyrifos. These results are supported by various reports on the effects of organophosphate pesticides on the liver of *O. niloticus*^64^; the gills and liver of *Catla catla*^65^ and the liver, gills and kidney of *Cyprinus carpio carpio*^66^. In most cells, peroxisome catalases are found, which are also known as oxidative biomarker enzymes that are involved in breaking down hydrogen peroxide (H_2_O_2_) into molecular oxygen (O_2_) and water, thereby neutralizing its harmful effects^67^. Superoxide radicals might be the reason for the decrease in CAT activity in the tissues, as they are known to inhibit CAT activity^68^. The oxidative damage to cells caused by chlorpyrifos might explain the reduction in catalase activity observed in this study.

One of the key enzymes that helps to protect the cells from oxidative stress by neutralizing superoxide and hydroxyl radicals, thereby counteracting oxygen toxicity, is Superoxide Dismutase (SOD). During this study, it was observed that as the concentration of CPF increased, SOD activity decreased. Noticed a significant decline in the SOD activity because of the disproportionation process, in which superoxide radicals are converted into hydrogen peroxide and oxygen^69^. A comparable finding of decreased SOD activity has been documented in *Oreochromis niloticus* and that were exposed to CPF^64^. Observed that the antioxidant responses of fish bodies are highly sensitive to environmental contamination and are frequently utilized in aquatic environmental health monitoring. Therefore, it is valuable to investigate oxidative stress and the dysregulation of antioxidant enzymes resulting from chlorpyrifos exposure in various organs. Furthermore, a significant reduction of SOD and CAT activities in liver, kidney, and gill tissues represents an adaptive response to protect the fish from free radical toxicity induced by insecticide.

These alterations are mostly caused by oxidative stress, which is a main mechanistic mechanism that is accountable for them. An excessive production of reactive oxygen species can lead to a depletion of cellular defence mechanisms, which can be shown by a large drop in antioxidant enzymes like superoxide dismutase and catalase. This is because these enzymes are responsible for destroying free radicals. On the other hand, the presence of concomitant elevations in hepatic transaminases (ALT and AST) is symptomatic of a damaged hepatocellular structure as well as a loss of membrane integrity. These biochemical markers of liver injury are strongly supported by histopathological data, which demonstrate vacuolar degeneration, cellular disintegration, and localised necrosis. This provides further evidence that the injury is not a temporary functional limitation but rather a cellular injury that is caused by oxidative stress^70^. When all the findings are considered together, they lend validity to a mechanistic theory that proposes that extended exposure to chlorpyrifos generates oxidative stress, which in turn causes progressive damage to metabolically active organs such as the liver and the gills. This dysfunction at the organ level finally shows as systemic haematological and metabolic abnormalities, which ultimately define an integrated pathophysiological response to long-term exposure to a pesticide that is not deadly.

Fish gills’ placement and large exterior area make them sensitive to various contaminants and pollutants present in water^71^. In several investigations, it has been shown that pesticides have similar effects on fish gills, which are responsible for a number of functions including respiration, digesting, osmoregulation and excretion. Some researchers documented that various gill histological changes were observed in different fish species exposed to different concentrations of various chemicals, which were similar to the outcomes of the present study^39,72^. A study documented that epithelial lifting, secondary lamellae fusion and hyperplasia stimulate the defense mechanism, which prevents contact between blood and the external pollutant environment^73^. Researcher reported that aneurysm might have occurred due to the toxic external environment, as a result, severe epithelial rupturing and haemorrhage were observed, which might be the reason for the present findings^74^. The secondary lamellae’s reduced ability to absorb oxygen as a result of the produced histological lesions in the gill tissues can also induce respiratory distress, which inhibits fish activity and growth.

In the case of chlorpyrifos exposure, the gill tissues of the fish exhibited potential implications for oxygen consumption and disruption of osmoregulatory activities. Fish gills are crucial for respiration and osmoregulation, and histological lesions, such as lamellar fusion and epithelial lifting, can reduce the efficiency of oxygen absorption by the secondary lamellae. This reduction in oxygen uptake can lead to respiratory distress, subsequently inhibiting fish activity and growth.

The liver is the primary organ for detoxifying metabolites, and its histology could be used to demonstrate the influence of toxicants at the cellular level^75^. In *O. niloticus*, a two-week exposure to chlorpyrifos resulted in increased degeneration, necrosis, and hemorrhage in liver tissue^75^. Degenerative hepatocytes, necrosis, vacuoles, vascular dilation, acute/chronic necrosis were noted on the liver tissue of Nile tilapia exposed to chlorpyrifos^39^. Also, the current study observation and the results of Sivkumar et al.^76^ report the same alteration in the liver tissue of *Clarias fuscus* exposed to monocrotophos. The liver’s purpose is to eliminate hazardous substances, but a high concentration of these compounds might impair its regulating systems, potentially resulting in structural damage^77^. The effects of an elevated CPF concentration in the liver may cause necrosis and degeneration in the hepatic cells because liver cells inability to regenerate new cells due to continuous exposure to toxicants.

The histological abnormalities identified in the liver during the present investigation point towards significant liver damage, consistent with findings in other studies involving exposure to various environmental contaminants. Understanding these alterations in liver tissue is crucial for assessing the impact of chlorpyrifos on the overall health and physiological functions of aquatic organisms.

The kidney is a vital organ, and its proper functioning is essential for maintaining homeostasis. The kidney is primarily involved in the elimination of waste from the blood and also performs selective reabsorption of certain ions that are essential for maintaining blood volume, pH, bodily fluids and erythropoieses^78^. In the present study, histological lesions were observed in the kidney of striped catfish exposed to chlorpyrifos. Similar results were found when common carp and Nile tilapia were exposed to indoxacarb and glyphosate, respectively^79,44^. Similar findings were also observed by Boran et al.^80^ in rainbow trout when exposed to different concentrations of captan. These histopathological lesions in the kidneys of chlorpyrifos treated fish might be due to impaired filtration flow and poor reabsorption process. Renal lesions can be considered as a trustworthy indicator of environmental pollution, as we know, the kidneys receive the largest portion of postbranchial blood. Disrupted glomerular filtration activity and impaired excretion may contribute to the buildup of toxicants in the kidney, leading to organ failure^81^. The histopathological findings in this study suggest that exposure to lethal concentrations of CPF resulted in detrimental effects on the gills, liver, and kidneys of *P. hypophthalmus*.

Based on the present study and prior research, the histopathological alterations observed in these tissues may stem from severe physiological problems, ultimately leading to the demise of fish. These findings underscore the importance of understanding the impact of environmental contaminants on the intricate physiological processes of aquatic organisms, emphasizing the need for effective conservation and pollution control measures.

## Conclusion

The outcomes of this study confirm the high toxicity of chlorpyrifos 20 EC to aquatic vertebrates by showing that sub-lethal exposure causes significant and consistent changes in haematological parameters, biochemical profile, enzymatic activities, and tissue architecture in *P. hypophthalmus* fingerlings. Anaemia, metabolic disruption, enzyme leakage, AChE inhibition, and histopathological damage have all been observed. These findings suggest that oxidative and physiological stress caused by chlorpyrifos can impair cellular function and possibly lead to illness or mortality under long-term exposure. Additionally, the integrated response of biochemical, enzymatic, histological, and haematological biomarkers highlights their utility as sensitive and trustworthy tools for early pesticide pollution detection and monitoring in aquaculture and natural waters. Chlorpyrifos levels reported in contaminated environments are significantly higher than the experimental concentrations used in this study (0.01 mg L^−1^ and 0.02 mg L^−1^), highlighting the pesticide’s potential ecological risk and practical relevance. The improper application of chlorpyrifos in agricultural landscapes next to aquatic systems is a major concern raised by these findings. Further research should focus on long-term, multi-stressor, and field-based assessments to better understand population-level impacts and to support the development of safer pesticide management strategies.

## Data Availability

Data will be available on request from corresponding author.
